# Predictors of pain and use of pain medications following primary Total Hip Arthroplasty (THA): 5,707 THAs at 2-years and 3,289 THAs at 5-years

**DOI:** 10.1186/1471-2474-11-90

**Published:** 2010-05-13

**Authors:** Jasvinder A Singh, David Lewallen

**Affiliations:** 1 Department of Health Sciences Research, Mayo Clinic College of Medicine, 200 First Street SW, Rochester, MN 55905, USA; 2 Department of Orthopedic Surgery, Mayo Clinic College of Medicine, 200 First Street SW, Rochester, MN 55905, USA; 3 Medicine Service, Birmingham VA Medical Center, Birmingham, 700 South 19th Street Birmingham, AL 35233, USA; 4 Department of Medicine, University of Alabama, Faculty Office Tower 805B, 510 20th Street South, Birmingham, AL 35294, USA

## Abstract

**Background:**

Study pain and use of pain medications and their predictors after primary Total Hip Arthroplasty (THA).

**Methods:**

We examined whether gender, age (reference, < = 60 yrs), body mass index (BMI; reference, <25 kg/m^2^)), comorbidity measured by Deyo-Charlson index (5-point increase), anxiety and depression predict moderate-severe hip pain and use of pain medications 2- and 5-years after primary THA. Multivariable logistic regression adjusted for these predictors and distance from medical center, operative diagnosis, American Society of Anesthesiologists (ASA) score and implant type.

**Results:**

Moderate-severe pain was reported by 8.1% at 2-years and 10.8% at 5-years. Significant predictors of moderate-severe pain at 2-year follow-up were [Odds ratio (95% confidence interval)]: BMI 35-39.9, 1.8 (1.2,2.8); BMI > = 40, 1.7 (1.0,2.9); depression, 2.1 (1.4,3.0). Moderate-severe pain at 5-years was more common in patients with higher BMI: 25-29.9, 1.5 (1.1,2.1); 30-34.9, 1.8 (1.2,2.6); 35-39.9, 1.9 (1.2,3.1); and > = 40, 3.1 (1.7,5.7).

Significant predictors of NSAID use were [Odds ratio (95% confidence interval)]: female gender at 2- and 5-years, 1.4 (1.1,1.7) and 1.4 (1.1,1.8); BMI 35-39.9 at 2-years, 1.9 (1.4, 2.6) and 30-34.9 at 2-years, 1.7 (1.2,2.4); and depression at 5-years, 1.8 (1.2,2.8).

Significant predictors of opioid medication use were [Odds ratio (95% confidence interval)]: female gender at 2- and 5-years, 2.0 (1.1,3.0) and 2.4 (1.4,4.0); BMI 30-34.9 at 2-years, 2.0 (1.0,3.9); and depression at 2-years, 2.0 (1.1,3.7).

**Conclusions:**

Higher BMI and depression impacted moderate-severe pain; and female gender, higher BMI and depression predicted use of pain medications at 2- and 5-years post-primary THA.

## Background

Primary Total Hip Arthroplasty (THA) is an extremely successful surgery [1] associated with significant pain relief and improvement in function and quality of life [2]. Implant survival studies indicate a very low failure rate of <1% per year [3,4]. While important clinical outcomes/complications such as fracture, infection and revision have been studied well, well-designed studies assessing patient-reported outcomes have been getting more attention in the last decade [5]. Pain is the number one reason for patients undergoing these procedures and therefore recurrence and/or persistence of significant hip pain after THA is of great concern for both patients and surgeons. While several important unmodifiable patient characteristics such as age and gender can impact pain outcomes following primary THA, it is important to know whether modifiable factors such as Body Mass Index and comorbidity impact pain following primary THA.

Are Body mass index (BMI) and comorbidity associated with pain outcomes after THA? The evidence is contradictory. BMI was associated with more pain at 6-12 months post-primary THA in one study [6], but not in others [7-9]. Comorbidity was found to be associated with pain outcomes post-THA in all studies [7,9-11] except one [6]. The main limitation of most previous studies is a small sample size that makes them underpowered to detect significant associations, thereby leading to false negative results. These studies also differed in measures of pain outcomes, length of follow-up and confounders adjusted in the analyses.

Non-modifiable factors of interest, such as gender have also been studied. Some studies have reported that female gender is associated with worse pain outcomes post-primary THA [7,9,12], while other studies found no gender association [10,11,13,14]. Similarly, younger age was associated with more pain 1-15 years post-THA [9,12-14], while others found no difference in pain outcomes by age, at 6-12 months [6,7,10,11,15]. A recent systematic review assessing prognostic factors for patient outcomes in THA studies up to 2001 reported that female gender was associated with less post-operative pain, after THA [16].

We hypothesized that at 2- and 5-years post-THA: (1) higher body mass index and comorbidity will be associated with worse pain outcomes; (2) pre-operative depression and anxiety, female gender and younger age will be associated with worse pain outcomes and higher use of pain medications.

## Methods

We used the data collected prospectively in the Mayo Total Joint Registry, detailed in previous studies [17,18]. Starting in 1993, all patients who underwent hip arthroplasty at the Mayo Clinic received a hip questionnaire that included questions regarding hip pain and function [19]. This validated questionnaire was administered at 2- and 5-years post-operatively by mail or in person at the clinical follow-up visit [20,21]. Patients who did not return the mailed survey and failed to return for follow-up clinic visits, had the questionnaire administered on the telephone by experienced registry staff. The study was approved by the Mayo Clinic Institutional Review Board. Several studies using these questionnaires and outcomes in knee and hip arthroplasty patients have been published or are in press [22-25]. The study cohort consisted of all patients who underwent primary THA at the Mayo Clinic, Rochester between 1993 and 2005 and were alive at the 2- or 5-year follow-up. We did not restrict by age group or diagnosis. A total of 9,154 THAs at 2- and 6,243 THAs at 5-year were eligible for the study.

### Outcomes of Interest

We assessed the prevalence of moderate-severe pain and the use of pain medications for THA pain at both 2- and 5-years post-primary THA. The pain question was: "How much pain do you have in your operated hip?" patients could respond- 'none', 'mild', 'moderate', 'severe'; none/mild was reference and moderate and severe categories were combined. A combined outcome of moderate-severe pain was chosen for several reasons: (1) the primary reason for undergoing THA is moderate-severe hip pain; and according to the clinical opinion of the senior orthropedic surgeon (D.L.) this was an undesirable outcome following THA, a major elective surgery; (2) this outcome was chosen *a priori*, before performing any data analyses except examining the prevalence of severe and moderate pain; and (3) even with a long follow-up duration of 13 years, very small number of patients had severe pain, making the multivariable analyses not meaningful.

Use of pain medications was assessed separately for NSAIDs and opioid medications. Patients were asked the following question "Do you use any of the following medications for the pain in your operated hip?" Patients could choose one or more responses from - 'none', 'Non-steroidal anti-inflammatory drugs (NSAIDs)', 'narcotics' or 'oral steroids'. We used none/oral steroid as the reference category and use of opioids and NSAIDs separately. This was based on the a priori determination by the senior orthopedic surgeon that the risks and implications of NSAIDs use are much different from the use of the opioid medications. Thus, clinically these outcomes are distinct. Oral steroids are used for reducing joint and/or systemic inflammation, usually but not always associated with significant pain and some patients may be chronically steroid-dependent. Additionally, only a small fraction of patients reported oral steroid use, making it impossible to analyze as a separate outcome.

### Predictors of Interest

The predictors of interest were categorized as: (1) unmodifiable - age and gender; and (2) modifiable - BMI, comorbidity, depression and anxiety. Age was categorized, as previously [22,26] into ≤ 60, 61-70, 71-80 and >80. BMI was categorized, as previously [27] into ≤ 25, 25.1-29.9, 30-34.9, 35-39.9 and ≥ 40. Comorbidity measured by Deyo-Charlson score, a validated comorbidity measure [28] was treated as a continuous variable, and we assessed the association of 5-point increase with outcomes. This is the most commonly used comorbidity measure consisting of a weighted scale of 17 comorbidities (including cardiac, pulmonary, renal, hepatic disease, diabetes, cancer, HIV etc.), expressed as a summative score [29,30]. Depression and anxiety were assessed by the presence or absence of International Classification of Diseases- ninth revision (ICD)-9 codes before the THA.

### Statistical Analyses

Baseline clinical and demographic characteristics of patients were compared using Student's t-test for continuous and chi-square tests for categorical measures. Responder and non-responder characteristics were compared using logistic regression analyses.

Univariate and multivariable-adjusted logistic regression analyses were performed for each outcome at 2- and 5-years. For these analyses, we used a Generalized Estimating Equations (GEE) approach to adjust for the correlation between observations on the same subject due to replacement of both hips. In addition to adjusting for primary predictors of interest -age, gender, BMI, comorbidity, anxiety and depression, all logistic regression analyses were also adjusted for the following potential confounders: (1) operative diagnosis- osteoarthritis, rheumatoid/inflammatory arthritis, avascular nerosis and other; (2) distance from the medical center- <100, 100-500 and >500 miles/overseas; (3) implant type- cemented, uncemented, hybrid; and (4) ASA class: Class I or II vs. III/IV. Distance from the medical center was included, since Mayo Clinic provides THA as a primary medical center for the local residents, but is also referral center for patients traveling from far, who may have different severity of disease and different level of expectation; both can impact pain outcomes [31]. We present only the multivariable-adjusted estimates for the ease of understanding. Odds ratios and the 95% confidence intervals are presented. The odds ratio defines the risk of an outcome in those with the risk factor compared to those without, such that a number >1 indicates higher occurrence of the outcome in those with each specific risk factor. A p-value < 0.05 and 95% confidence interval excluding one indicates statistical significance.

Each author certifies that his or her institution has approved the human protocol for this investigation and that all investigations were conducted in conformity with ethical principles of research.

## Results

### Characteristics of the study population and the non-responders

The characteristics of responders at 2- and 5-years were similar, as described in Table 1. The survey response rate was 62.3% (5,707/9,154) at 2- and 52.7% (3,289/6,243) at 5-years post-primary THA. In this study, we studied 5,707 primary THAs at 2-years and 3,289 primary THAs at 5-years. At 2-years post-primary THA, the mean age of responders was 65 years, 51% were female, 30% were ≤60 years, 24% had BMI <25 kg/m^2^, 35% had an uncemented implant and the underlying diagnosis was osteoarthritis in 87% (Table 1). The response rate was 62.3% (5,707/9,154) at 2-years and 52.7% (3,289/6,243) at 5-years post-primary THA. 95% of patients each from 2-year and 5-year follow-up cohorts reported moderate-severe pain in the index hip at pre-surgery evaluation.

**Table 1:** Characteristics of patients

	Primary THA	
	
	2-year (n = 5,707)	5-year (n = 3,289)
Mean Age (± SD)	65.0 ± 13.3	64.7 ± 12.9
Men/Women (%)	49%/51%	47%/53%
**Age groups n (%)**		
≤ 60 yrs	30%	30%
>60-70 yrs	31%	32%
>70-80 yrs	30%	31%
>80 yrs	9%	6%
**Body Mass index **(in kg/m^2^)		
≤ 24.9	24%	24%
25-29.9	39%	40%
30-34.9	24%	23%
35-39.9	8%	8%
≥ 40	4%	4%
Missing	1%	1%
**ASA Score**		
Class I-II	62%	64%
Class III-IV	38%	36%
Missing	1%	1%
**Cemented**		
Yes	11%	14%
Hybrid	54%	59%
Uncemented	35%	27%
**Underlying Diagnoses**		
Rheumatoid Arthritis/Other Inflammatory arthritis conditions	3%	3%
Osteoarthritis	87%	85%
Avascular Necrosis	7%	8%
Other^a^	4%	4%

^a^Other category includes congenital dislocated hip, neoplasm, fracture, Legg-Calvé-Perthe's etc.

Survey responders at 2-years post-primary THA were more likely to be older (age 61-70, 71-80 and >80 with odds ratios (OR), 1.4, 1.3 and 1.3, compared to ≤ 60 years) and less likely to have BMI ≥ 40 (OR, 0.7), ASA class III-IV (OR, 0.9) or live farther from the medical center (distance >100-500 and >500 miles with OR 0.9 and 0.7). At 5-years, responders were more likely to be older (age 61-70, 71-80 with OR, 1.4 and 1.4), BMI 25-29.9 (OR, 1.2), and less likely to live farther from the medical center (distance >100-500 and >500 miles with OR 0.9 and 0.6).

### Prevalence of pain and use of pain medications after THA

Moderate-severe pain was reported by 8.1% (435/5,390) at 2-years and 10.8% (339/3,130) at 5-years. NSAID use was reported by 12% (607/5,064) at 2- years and 12.7% (382/3,005) at 5-years. Opioid medication use was reported by 2.3% (118/5,064) at 2- years and 2.8% (85/3,005) at 5-years.

### Predictors of Moderate-severe pain after Primary THA

Two years after primary THA, we noted significantly higher odds of moderate-severe hip pain in patients with BMI 35-39.9 and ≥ 40 with odds of 1.8 and 1.7, compared to BMI <25; and presence of depression increased the odds of moderate-severe pain to 2.1, compared to those without depression (Table 2). Gender, age, comorbidity and presence of anxiety were not significantly associated with hip pain.

**Table 2:** Multivariable* Predictors of Moderate-severe Pain

	Multivariable-adjusted 2-year			Multivariable-adjusted 5-year		
	**Odds Ratio**	**(95% Confidence Interval)**	**p-value**	**Odds Ratio**	**(95% Confidence Interval)**	**p-value**

**Female Gender (Ref**, Male)	1.1	(0.9, 1.4)	0.30	1.2	(0.9, 1.5)	0.18
**Age (Ref**, ≤ 60 yrs)						
>60-70 yrs	0.7	(0.6, 1.0)	0.06	0.8	(0.6, 1.1)	0.17
>70-80 yrs	1.1	(0.8, 1.6)	0.45	0.8	(0.6, 1.2)	0.32
>80 yrs	1.5	(1.0, 2.3)	0.07	0.6	(0.3, 1.1)	0.11
**BMI (Ref**, <25 kg/m^2^)						
25-29.9	1.0	(0.7, 1.3)	1.00	**1.5**	**(1.1, 2.1)**	**0.02**
30-34.9	1.3	(1.0, 1.8)	0.07	**1.8**	**(1.2, 2.6)**	**<0.01**
35-39.9	**1.8**	**(1.2, 2.6)**	**<0.01**	**1.9**	**(1.2, 3.1)**	**0.01**
≥ 40	**1.7**	**(1.0, 2.9)**	**0.04**	**3.1**	**(1.7, 5.7)**	**<0.01**
**Deyo-Charlson index (**5-point change)	1.0	(0.8, 1.3)	0.98	0.9	(0.7, 1.3)	0.75
Anxiety **(Ref**, no)	0.9	(0.5, 1.4)	0.58	1.1	(0.6, 2.1)	0.76
Depression **(Ref**, no)	**2.1**	**(1.4, 3.0)**	**<0.01**	1.3	(0.8, 2.2)	0.30

*Adjusted for ASA score, distance from the medical center, income and the operative diagnosis, in addition to the above variables

Regression: n/N = 409/5,154 at 2-yr FU; n/N = 318/2,929 at 5-yr FU; **Numbers in Bold indicate significant Odds ratios and p-values**

At 5-years post-primary THA, all BMI categories above 25 were significantly associated with higher odds of moderate-severe hip pain, with increasing odds with increasing BMI (Table 2). Gender, age, comorbidity, anxiety and depression were not significantly associated.

### Predictors of Use of NSAID and Opioid Pain medications after Primary THA

#### NSAID use

At 2-years, NSAID use was significantly higher in women with odds of 1.4, in patients with BMI of 35-39.9 with odds of 1.9 and in patients with depression with odds of 2.0 (Table 3). At 5-years, women had 1.4 times odds of NSAID use and those with BMI 30-34.9 kg/m^2^, 1.7 times.

**Table 3:** Multivariable* Predictors of Use of NSAID medications

	Multivariable-adjusted 2-year			Multivariable-adjusted 5-year		
	
	Odds Ratio	(95% Confidence Interval)	p-value	Odds Ratio	(95% Confidence Interval)	p-value
**Female Gender (Ref**, Male)	**1.4**	**(1.1, 1.7)**	**<0.01**	**1.4**	**(1.1, 1.8)**	**<0.01**
**Age (Ref**, ≤ 60 yrs)						
>60-70 yrs	1.1	(0.9, 1.4)	0.34	0.9	(0.7, 1.3)	0.70
>70-80 yrs	1.3	(0.9, 1.7)	0.12	0.9	(0.6, 1.3)	0.49
>80 yrs	1.4	(0.9, 2.0)	0.12	1.1	(0.6, 1.9)	0.79
**BMI (Ref**, <25 kg/m^2^)						
25-29.9	1.0	(0.8, 1.3)	0.99	1.4	(1.0, 1.8)	0.06
30-34.9	1.2	(0.9, 1.5)	0.28	**1.7**	**(1.2, 2.4)**	**<0.01**
35-39.9	**1.9**	**(1.4, 2.6)**	**<0.01**	1.5	(1.0, 2.4)	0.07
≥ 40	**1.6**	**(1.0, 2.5)**	**0.04**	**1.9**	**(1.1, 3.4)**	**0.03**
**Deyo-Charlson index (**5-point change)	1.0	(0.7, 1.3)	0.81	1.1	(0.8, 1.5)	0.58
**Anxiety (Ref**, no)	1.2	(0.8, 1.8)	0.46	0.8	(0.5, 1.5)	0.56
**Depression (Ref**, no)	1.1	(0.8, 1.6)	0.52	**1.8**	**(1.2, 2.8)**	**<0.01**

*Adjusted for ASA score, distance from the medical center, income and the operative diagnosis, in addition to the above variables

Regression: n/N = 575/4,830 at 2-yr FU; n/N = 362/2,807 at 5-yr FU; **Numbers in Bold indicate significant Odds ratios and p-values**

#### Opioid medication use

Two years after primary THA, women had odds of 2.0, and those with depression odds of 2.0, of using opioid medications (Table 4). Five years after primary THA, opioid medication use was significantly higher in women with odds of 2.4 and in patients with BMI 30-34.9 kg/m^2 ^with odds of 2.0.

**Table 4:** Multivariable* Predictors of use of opioid medications

	Multivariable-adjusted 2-year			Multivariable-adjusted 5-year		
	**Odds Ratio**	**(95% Confidence Interval)**	**p-value**	**Odds Ratio**	**(95% Confidence Interval)**	**p-value**

**Female Gender (Ref**, Male)	**2.0**	**(1.3, 3.0)**	**<0.01**	**2.4**	**(1.4, 4.0)**	**<0.01**
**Age (Ref**, ≤60 yrs)						
>60-70 yrs	0.9	(0.5, 1.5)	0.71	0.7	(0.3, 1.3)	0.25
>70-80 yrs	0.7	(0.4, 1.3)	0.22	0.8	(0.4, 1.7)	0.63
>80 yrs	1.0	(0.5, 2.3)	0.93	1.1	(0.4, 3.3)	0.84
**BMI (Ref**, <25 kg/m^2^)						
25-29.9	0.9	(0.6, 1.5)	0.79	1.5	(0.8, 2.8)	0.17
30-34.9	1.3	(0.8, 2.2)	0.29	**2.0**	**(1.0, 3.9)**	**0.04**
35-39.9	1.0	(0.5, 2.3)	0.91	0.7	(0.2, 2.4)	0.53
≥ 40	1.4	(0.6, 3.6)	0.45	1.3	(0.4, 4.9)	0.65
**Deyo-Charlson index (**5-point change)	1.3	(0.8, 2.1)	0.25	1.5	(0.8, 2.7)	0.18
**Anxiety (Ref**, no)	0.8	(0.3, 1.9)	0.60	2.0	(0.8, 5.0)	0.14
**Depression (Ref**, no)	**2.0**	**(1.1, 3.7)**	**0.02**	1.4	(0.6, 3.4)	0.42

*Adjusted for ASA score, distance from the medical center, income and the operative diagnosis, in addition to the above variables

Regression: n/N = 114/4,830 at 2-yr FU; n/N = 78/2,807 at 5-yr FU

Numbers in Bold indicate significant Odds ratios and p-values

## Discussion and conclusions

In this study, we found that higher body mass index was associated with significantly higher odds of moderate-severe hip pain and use of NSAID medications 2-years after primary THA. At 2-years post-primary THA, female gender was associated with higher odds of NSAID and opioid medication use. Depression was associated with higher odds of moderate-severe pain and opioid medication use at 2-years post-primary THA. Most of these associations were still significant at 5-years.

Our study has many novel findings that merit further discussion. We believe our study is among the first to provide the estimates of NSAIDs and opioid medication use after primary THA in a large sample of patients. Despite several studies of pain after THA, quite surprisingly, the prevalence of pain medication use at intermediate- or long-term follow-up after THA has not been described. In this large sample of patients, we provide estimates of moderate-severe pain at intermediate-term follow-up after primary THA. This information can be provided at pre-operative counseling so that patients have realistic expectations from the procedure. Studies are needed to examine use of these mediations at long-term follow-up after THA.

Second, depression predicted moderate-severe pain and higher opioid medication use 2-years post-primary THA. Previous studies in primary knee arthroplasty patients have reported that depression predicts pain at 1- [32], 2- [33] and 5-years [34]. To our knowledge, there are no published studies that have examined whether depression is associated with pain or pain medication use after primary THA. Our findings suggest that screening for depression prior to surgery may help identify patients at-risk for poorer pain outcomes and opioid use 2-years after THA. A potential explanation of association of baseline depression with 2-year outcomes may be higher pain perception and reporting by those with depression than those without depression. Further studies need to examine if treatment of depression in the pre- or post-operative period improves pain outcomes and opioid medication use 2-years after THA. At 5-years, these associations were not significant. The lack of significance with 5-year outcomes may be due to smaller sample size at 5-years compared to 2-years, longer duration since depression diagnosis or simple lack of any association at 5-years.

The third important finding in this study is that higher BMI significantly predicted a higher risk of moderate-severe hip pain at 2- and 5-years. This finding confirms a similar finding in one other study of 193 patients at 6-12 months post-primary THA [6], but is in contrast to three multivariable-adjusted studies that found no such association at 0.5-years [7] or at 15-years [8,9]. All previous studies were multivariable-adjusted, similar to our study. Previous studies had examined BMI as a continuous [6,7] or three-level categorical variable (<25, 25-30, >30) [8,9], compared to 5-level categorical variable in our study, as per the WHO classification [27]. The main reason for two of the previous studies being negative was small sample size, making them liable to type II error, i.e., missing an effect when one exists due to a small sample size. We categorized BMI as per WHO classification [27], since our sample size was large enough to not restrict us to fewer categories. Another interesting observation was a dose-relationship of increasing BMI with moderate-severe pain at 5-year follow-up.

Fourth, the association of female gender with NSAID use and opioid medication use at 2- and 5-years, but not moderate-severe pain merits some discussion. Lack of gender-pain associations in our study is similar to many studies [10,11,13,14], but in contrast to others [7,9,12]. Higher odds of use of NSAIDs and opioid medications in women versus men with primary THA is similar to higher analgesic use reported for women in national cohorts of patients in US and Sweden [35,36]; our study extends this finding to THA cohorts. Our finding of lack of gender and pain severity in THA patients is also interesting, since we have reported higher prevalence of pain in women at 2- and 5-year post-TKA from the same dataset [22]. Thus, gender has different impact on pain outcomes after THA versus TKA.

Lastly, the lack of association of older age with pain outcomes confirmed similar findings in studies of primary THA [6,7,10,11], in contrast to others [9,12-14]. Again, it is interesting that age associations we noted in post-TKA outcomes from the same dataset were not true for post-THA [22].

Our study has several strengths. We examined the NSAID and opioid medication use alongside pain assessment at both 2- and 5-years after primary THA in a large cohort. This is the most important contribution of this study. Most previous studies were limited to 1-2 year follow-up and had not included study of use of pain medications for residual pain after THA. The study of use of pain medications is an important outcome, which is under-studied in the arthroplasty literature. We controlled for several important confounders and covariates and our estimates of association were robust i.e., changed very little between univariate and multivariable models.

Our study has several limitations. Non-response and referral bias may limit generalizability to general populations. The response rate of 62% at 2-years and 53% at 5-years, is similar to the average response rate of 60% in large surveys of this size [37]. A higher response rate is always more desirable. The estimates of association in this study are conservative, since non-responders were more likely to be younger, have ASA class III-IV and live >100 miles away from the medical center and the same categories that also reported more pain. Residual confounding is possible in this cohort study due to our inability to control for pre-operative pain in the main analyses, since this would have led to selection bias with only less than half subjects eligible in each category. We were unable to adjust for acute pain management after THA, which may impact chronic postoperative pain [38]. Depression and anxiety would be best captured by examination by a psychologist or use of validated instruments such as Center for Epidemiological Studies Depression (CES-D) scale or Beck's Depression Inventory. This was a retrospective analysis of prospectively collected data of all-comers for THA over a 12-year period that did not include depression questionnaires; and psychiatric evaluation of every patient undergoing THA is neither feasible nor clinically appropriate. Currently there is no National U.S. Joint Registry, and therefore an analysis of data collected over a considerable time-period from a large volume medical center (such as this) is the next best approach. Future studies can examine the predictors of moderate and severe categories separately, if it is clinically indicated.

In summary, we studied pain severity and use of pain medications 2- and 5-years after primary THA in a large cohort of patients. We noted significant impact of BMI on moderate-severe pain and use of pain medications 2- and 5-years after THA. Associations with depression with moderate-severe pain were significant at 2-years, but not at 5-year follow-up. Female gender was associated with more pain medication use, but similar pain outcome. This study identifies modifiable factors for pain outcomes, which can be targeted with pre- or post-operative interventions in future studies.

## Competing interests

One of the authors (DL) has received royalties/speaker fees from Zimmer, has been a paid consultant to Zimmer and has received institutional research funds from DePuy, Stryker and Zimmer. J.A.S. has received speaker honoraria from Abbott; research and travel grants from Allergan, Takeda, Savient, Wyeth and Amgen; and consultant fees from Savient and URL pharmaceuticals.

## Authors' contributions

JAS- Study concept and design, Ethics committee approval, Data analysis plan, Data analyses, Preparation and revision of manuscript. SA- Study concept and design, Data analysis plan, preparation and revision of manuscript. Both authors read and approved the final manuscript.

## Pre-publication history

The pre-publication history for this paper can be accessed here:

http://www.biomedcentral.com/1471-2474/11/90/prepub
